# Supplementary material for: Systematic review to evaluate accuracy studies of the diagnostic criteria for periodontitis in pregnant women
Source: PLoS One. 2024 Jul 17;19(7):e0304758. doi: 10.1371/journal.pone.0304758 (PMC11253960; doi:10.1371/journal.pone.0304758)
Supplement: S1 File — (DOCX) [file pone.0304758.s002.docx]

**S1 Chart 1.** Search strategies with uniterms and Boolean operators used according to electroncal databases.

| **MedLine/PUBMED (30/03/2024)** | **Total** |
| --- | --- |
| (((((((((((Pregnant Women[MeSH Terms]) OR (Pregnant Woman[Title/Abstract])) OR (Woman, Pregnant[Title/Abstract])) OR (Women, Pregnant[Title/Abstract])) OR (Pregnancy Complications[MeSH Terms])) OR (Pregnancy Complications[Title/Abstract])) OR (Pregnant Women[Title/Abstract])) OR (Complication, Pregnancy[Title/Abstract])) OR (Complications, Pregnancy[Title/Abstract])) OR (Pregnancy Complication[Title/Abstract])) AND (((((((((((((Periodontitis[MeSH Terms]) OR (Periodontitis[Title/Abstract])) OR (Pericementitides[Title/Abstract])) OR (Pericementitis[Title/Abstract])) OR (Periodontitides[Title/Abstract])) OR (Periodontal Diseases[MeSH Terms])) OR (Periodontal Diseases[Title/Abstract])) OR (Disease, Periodontal[Title/Abstract])) OR (Diseases, Periodontal[Title/Abstract])) OR (Parodontoses[Title/Abstract])) OR (Parodontosis[Title/Abstract])) OR (Periodontal Disease[Title/Abstract])) OR (Pyorrhea Alveolaris[Title/Abstract]))) AND (((((sensitiv*[Title/Abstract]) OR (sensitiv*[MeSH Terms])) OR (predictive value*[MeSH Terms])) OR (predictive value*[Title/Abstract])) OR (accurac*[Text Word])) | 37 |
| **LILACS** |  |
| (tw:((tw:(Pregnant Women)) OR (tw:(Woman, Pregnant)) OR (tw:(Women, Pregnant)) OR (tw:(Pregnancy Complications)) OR (tw:(Pregnant Women)) OR (tw:(Complication, Pregnancy)) OR (tw:(Complications, Pregnancy)) OR (tw:(Pregnancy Complication)))) AND (tw:((tw:(Periodontitis)) OR (tw:(Pericementitides)) OR (tw:(Pericementitis)) OR (tw:(Periodontitides)) OR (tw:(Periodontal Diseases)) OR (tw:(Disease, Periodontal)) OR (tw:(Diseases, Periodontal)) OR (tw:(Parodontoses)) OR (tw:(Parodontosis)) OR (tw:(Periodontal Disease)) OR (tw:(Pyorrhea Alveolaris)))) AND (tw:((tw:(sensitiv*)) OR (tw:(predictive value*)) OR (tw:(accurac*)))) | 8 |
| **WEB OF SCIENCE** |  |
| #4 1 AND #2 AND #3#1 TOPIC: (Pregnant Women) OR TOPIC: (Pregnant Woman) OR TOPIC: (Woman, Pregnant) OR TOPIC: (Women, Pregnant) OR TOPIC: (Pregnancy Complications) OR TOPIC: (Complication, Pregnancy) OR TOPIC: (Pregnancy Complication) #2 TOPIC: (periodontitis) OR TOPIC: (Pericementitides) OR TOPIC: (Pericementitis) OR TOPIC: (Periodontitides) OR TOPIC: (Periodontal Diseases) OR TOPIC: (Disease, Periodontal) OR TOPIC: (Diseases, Periodontal) OR TOPIC: (Parodontoses) OR TOPIC: (Parodontosis) OR TOPIC: (Periodontal Disease)  #3 TOPIC: (sensitiv*) OR TOPIC: (predictive value*) OR TOPIC: (accurac*) | 25 |
| **SciELO** |  |
| ((pregnant women) OR (pregnant woman) OR (woman, pregnant) OR (pregnancy complications) OR (complication, pregnancy) OR (complications, pregnancy) OR (pregnancy complication)) AND ((periodontitis) OR (pericementitides) OR (periodontal diseases) OR (diseases, periodontal) OR (parodontoses) OR (parodontosis) OR (periodontal disease) OR (pyorrhea alveolaris)) | 25 |
| **Embase** |  |
| #4 1 AND #2 AND #3 #1 pregnancy:ti,ab,kw OR 'pregnant woman':ti,ab,kw  #2 periodontitis:ti,ab,kw OR 'periodontal disease':ti,ab,kw OR 'chronic periodontitis':ti,ab,kw  #3 sensitiv*:ti,ab,kw OR 'predictive value*':ti,ab,kw OR accurac*:ti,ab,kw | 40 |
| **SCOPUS** |  |
| ( ( TITLE-ABS-KEY ( "Pregnant Women" ) OR TITLE-ABS-KEY ( "Woman, Pregnant" ) OR TITLE-ABS-KEY ( "Women, Pregnant" ) OR TITLE-ABS-KEY ( "Pregnancy Complications" ) OR TITLE-ABS-KEY ( "Complication, Pregnancy" ) OR TITLE-ABS-KEY ( "Complications, Pregnancy" ) OR TITLE-ABS-KEY ( "Pregnancy Complication" ) ) ) AND ( ( TITLE-ABS-KEY ( periodontitis ) OR TITLE-ABS-KEY ( pericementitis ) OR TITLE-ABS-KEY ( pericementitis ) OR TITLE-ABS-KEY ( periodontitis ) OR TITLE-ABS-KEY ( "Periodontal Diseases" ) OR TITLE-ABS-KEY ( "Disease, Periodontal" ) OR TITLE-ABS-KEY ( "Diseases, Periodontal" ) OR TITLE-ABS-KEY ( parodontosis ) OR TITLE-ABS-KEY ( parodontosis ) OR TITLE-ABS-KEY ( "Periodontal Disease" ) OR TITLE-ABS-KEY ( "Pyorrhea Alveolaris" ) ) ) AND ( ( TITLE-ABS-KEY ( sensitiv* ) OR TITLE-ABS-KEY ( predictive AND value* ) OR ALL ( accurac* ) ) ) | 73 |
| **ProQuest** |  |
| ((Pregnant Women) OR (Pregnant Woman) OR (Woman, Pregnant) OR (Women, Pregnant)) AND (periodontitis OR Pericementitides OR Pericementitis OR Periodontitides OR (Periodontal Diseases) OR (Disease, Periodontal) OR (Diseases, Periodontal) OR Parodontoses OR Parodontosis OR (Periodontal Disease)) AND (sensitiv* OR (predictive value*) OR accurac*) | 370 |
| **CINAHL** |  |
| (TI sensitiv* OR AB sensitiv* OR TI predictive value* OR AB predictive value* OR TI accurac* OR AB accurac*) AND (S1 AND S2 AND S3)  #S1 TI Pregnant Women OR AB pregnant women OR TI Pregnant Woman OR AB Pregnant Woman OR TI Woman, Pregnant OR AB Woman, Pregnant OR TI Women, Pregnant OR AB Women, Pregnant OR TI Pregnancy Complications OR AB Pregnancy Complications OR TI Pregnancy Complication OR AB Pregnancy Complication  #S2 TI periodontitis OR AB periodontitis OR TI Periodontal Diseases OR AB Periodontal Diseases OR TI Disease, Periodontal OR AB Disease, Periodontal OR TI Diseases, Periodontal OR AB Diseases, Periodontal OR TI Parodontoses OR AB Parodontoses OR TI Periodontal Disease OR AB Periodontal Disease  #S3 (TI sensitiv* OR AB sensitiv* OR TI predictive value* OR AB predictive value* OR TI accurac* OR AB accurac*) | 14 |
