# Supplementary material for: Systematic review to evaluate accuracy studies of the diagnostic criteria for periodontitis in pregnant women
Source: PLoS One. 2024 Jul 17;19(7):e0304758. doi: 10.1371/journal.pone.0304758 (PMC11253960; doi:10.1371/journal.pone.0304758)
Supplement: S2 File — (DOCX) [file pone.0304758.s003.docx]

**S2 Chart 2.** Studies included in the systematic review to evaluate the accuracy of diagnostic criteria for periodontitis in pregnant women.

| **Authors**  **Year of publication Country of study Journal Published** | **Objective /**  **Funding sources** | **Study Design /**  **Sample size** | **Gold standard criteria used for diagnosing periodontitis** | **Index test criteria used for diagnosing periodontitis** | **Periodontitis frequency (gold standard)** | **Periodontitis frequency (index test)** | **Diagnostic values (sensitivity, specificity, predictive values, likelihood ratio) /**  **95% Confidence Interval (95%CI)** |
| --- | --- | --- | --- | --- | --- | --- | --- |
| CONCEIÇÃO et al.,  2023  Bahia and Pernambuco– Brazil  ***Caderno Pedagógico*** | Validate diagnostic criteria for periodontitis in pregnant women  **Funding sources**: Government institutions. | Cross-sectional study  **Sample**: 1251 pregnant women | Diagnosis according to  **Gomes-Filho et al. 2018**:  1. Severe periodontitis  ≥ 2 teeth with ≥ 1 sites with Probing Depth (PS) ≥ 5 mm, with Clinical Attachment Level  (CAL) ≥ 5 mm and bleeding on probing in the same CAL.  2. Moderate periodontitis: ≥ 2 teeth with ≥ 1 sites with PS ≥ 4 mm, with CAL ≥ 3 mm  and bleeding on probing at the same site 3. Mild periodontitis: ≥ 2 teeth with ≥ 1 sites with PS ≥ 4 mm, with CAL ≥ 1 mm and bleeding on probing at the same site.  4. No periodontitis: absence of “mild” or “moderate” or “severe” periodontitis.  Diagnosis according to | Diagnosis according to  **Centers for Disease Control and Prevention/American Academy of Periodontology (CDC/AAP)** **CRITERION I:**  1. Severe periodontitis: ≥ 2 interproximal sites with  (CAL) ≥ 6 mm (in different teeth) and ≥ 1 interproximal site with probing depth (PS) ≥ 5 mm.  2. Moderate periodontitis:  ≥ 2 interproximal sites with CAL ≥ 4 mm (in different teeth) or ≥ 2 interproximal sites with PS ≥ 5 mm. 3. Mild periodontitis: ≥2 interproximal sites with CAL ≥ 3 mm (not on the same tooth) and ≥2 interproximal sites with PS ≥4 mm (not on the same tooth) or 1 site with PS ≥ 5 mm.  4. No periodontitis: no “mild” or “moderate” or “severe” periodontitis  Diagnosis according to  **Albandar et al. 2007 CRITERION 2:**  1. Severe periodontitis  ≥ 2 teeth with ≥ 1 sites with PS ≥ 5 mm, with  CAL ≥ 5 mm.  2. Moderate periodontitis: ≥ 2 teeth with ≥ 1 PS sites  ≥ 4 mm and CAL ≥ 5 mm. 3. Mild periodontitis: ≥ 1 tooth with ≥ 1 sites with PS ≥ 4 mm and CAL ≥ 4 mm.  4. No periodontitis: absence of “mild” or “moderate” or “severe” periodontitis.  Diagnosis according to  **Bassani et al. 2007 CRITERION 3:**  1. Severe periodontitis  ≥ 3 interproximal  locations (on different teeth)  with CAL ≥ 7 mm. 2. Moderate periodontitis: ≥ 3 interproximal sites (in  different teeth) with CAL 5 mm. 3. Mild periodontitis: ≥ 3  interproximal sites (in  different teeth)  with CAL ≥ 3  mm. 4. No periodontitis: absence of “mild” or “moderate” or “severe” periodontitis.  Diagnosis according to  **Lopez et al. 2002 CRITERION IV:**  - Periodontitis: ≥ 4 teeth with ≥ 1 sites with PS  ≥ 4 mm, and with CAL ≥ 3  mm on a website.  Diagnosis according to  **Nesse et al. 2008 CRITERION V:**  - Periodontitis: Individuals  with the highest Periodontal Inflamed Surface area (PISA) quartile  amount. | **Occurrence of periodontitis:**  Gomes-Filho et al. 2018: 23.1.% (n=289) | **Occurrence of periodontitis:**  CDC/AAP: 41.1% (n=515)  Albandar et al. 2007:  39.7 % (n=465)  Bassani et al. 2007: 67.1% (n=840)  Lopez et al. 2002: 18.8%  (n=237)  Nesse et al. 24.8% (n=300) | (CDC/AAP) - CRITERION I  **Sensitivity**: 86.5% (82.0-90.0)  **Specificity**: 72.50% (69.5-75.3)  **Positive predictive value**: 48.5% (44.1- 53.0)  **Negative predictive value**: 94.7% (92.8-96.2)  **Positive likelihood ratio**: 3.1 (2.8- 3.5)  **Negative likelihood ratio**: 0.19 (0.14-0.25)  Albandar et al. 2007 -CRITERION 2  **Sensitivity**: 86.5% (82.0-90.2)  **Specificity**: 79.9% (77.3-82.4)  **Positive predictive value**: 58.5% (53.9-63.0)  **Negative predictive value**: 97.8% (96.8-96.7)  **Positive likelihood ratio**: 15.8 (4.6- 5.3)  **Negative likelihood ratio**: 0.07 (0.05-0.12)  Bassani et al. 2007 -CRITERION III  **Sensitivity**:98.6% (96.5-99.6)  **Specificity**:42.3% (39.2-45.5)  **Positive predictive value**:33.9% (30.7-33.2)  **Negative predictive value**:99.0% (97.5-99.7)  **Positive likelihood ratio**: 1.71 (1.62-1.81)  **Negative likelihood ratio**: 0.03 (0.01-0.09)  Lopez et al. 2002- CRITERION IV  **Sensitivity**: 70.6% (65.0-75.8)  **Specificity**: 96.8% (95.5-97.8)  **Positive predictive value:** 86.8% (89.7-93.3)  **Negative predictive value**: 91.6% (48.5-49.0)  **Positive likelihood ratio**: 21.8 (15.3-31.1)  **Negative likelihood ratio**:0.30 (0.25-0.36)  Nesse et al. 2008- CRITERION V  **Sensitivity**: 76.5% (71.1-81.2)  **Specificity**: 90.6% (88.6-92.4)  **Positive predictive value**:71.1% (65.7-76.0)  **Negative predictive value**: 92.8% (90.9-94.3)  **Positive likelihood ratio**: 8.1 (6.6-10.0)  **Negative likelihood ratio**: 0.26 (0.21-0.32) |
| Li et al,  2022  Shenzhen, China  Journal Peridontal Research | To compare the profile of periodontal diseases among pregnant women using the CDC/AAP and American Academy of Periodontics and the European Federation of Periodontics (AAP/EFP) systems and attempted to develop a clinically applicable and easy-to-use tool for screening maternal periodontal diseases by general dentists. | Cross-sectional study  **Sample**: 204 pregnant women | Diagnosis according to  (CDC/AAP):  1. Severe periodontitis: ≥ 2 interproximal sites with  CAL ≥ 6 mm (in different teeth) and ≥ 1 interproximal site with probing depth (PS) ≥ 5 mm.  2. Moderate periodontitis:  ≥ 2 interproximal sites with CAL ≥ 4 mm (in different teeth) or ≥ 2 interproximal sites with PS ≥ 5 mm.  3. All subjects without severe or moderate periodontitis were defined as mild periodontitis or no periodontitis, respectively. | **Diagnosis according to**  **(AAP/EFP):**  1. Periodontitis: CAL at ≥2 non-adjacent teeth, or buccal/oral CAL ≥3 mm with PD >3 mm at ≥2 teeth.  PS: As the radiographic examination was not performed in this study, grading was not given in the final classification of periodontitis.  2. Gingivitis: presence of gingival inflammation (Bleeding on probing- BOP ( ≥10% of sites) and the absence of detectable CAL due to periodontitis.  3. Periodontal health was defined as the absence of gingival inflammation (BOP <10% of sites) and the absence of CAL resulting from periodontitis  For staging,  Mild periodontitis (Stage I): based on interdental CAL 1-2 mm at the most affected sites.  Moderate periodontitis (Stage II): based on interdental CAL 3-4 mm at the most affected sites  Severe periodontitis (Stages III and IV): based on interdental  CAL ≥5 mm at the most affected sites  Diagnosis according **FDI Periodontal Diseases Chairside Guide (FDI-CG)**  1. The 7-item scoring system of FDI-CG for periodontal disease profiling consists of age, tobacco smoking, diabetes mellitus, tooth loss due to periodontitis, plaque deposits, BOP, and PD. Each item is ranged from 0 to 2 or 3, and the total score is then calculated accordingly.  2. Individual profile of periodontal diseases is then categorized into three levels accordingly, namely Mild (0–5), Moderate (6–10), and Severe (≥ 11) | **CDC/APP**  No &Mild: 69.1% (n=141)  Moderate: 30.4% (n= 62)  Severe: 55.0% (n=1) | **AAP/EFP:**  Healthy: 22.5% (n=46)  Gingivivitis: 19.6% (n=40)  Stage I: 22.1% (n=45)  Stage II: 23.5% (n=48)  Stage III: 12.5% (n=25)  Stage IV: 0  **FDI-CG**  Mild (0–5): 93.1% (n=190)  Moderate (6–10): 6.9% (n=14)  Severe (>10): 0.0% (n=0) | AAP/EFP:  **Sensitivity**: 100.0% (94.3-100.0)  **Specificity**: 92.9% (87.4-96.1)  **Positive predictive value:** 86.3% (76.6.7-92.4)  **Negative predictive value**: 100.0% (97.2-100)  FDI-CG original:  **Sensitivity**: 20.6% (12.5-32.2)  **Specificity**: 99.3% (96.1-100)  **Positive predictive value: 92.3**% (68.5-99.6)  **Negative predictive value**: 73.7% (67.0-74.9)  Version was that pregnancy would be scored with 2 additional points **(FDI-CG all +2):**  **Sensitivity**: 100% (94.3-100)  **Specificity**: 61.7% (53.5-69.3)  **Positive predictive value:** 53.9% (44.8-62.6)  **Negative predictive value**: 100% (95.7-100)  Was that early phase of pregnancy was scored with 1 additional point, and late phase of pregnancy was given 2 additional points (**FDI-CG early phase +1 and late phase +2**):  **Sensitivity**: 92.1% (82.7-96.6)  **Specificity**: 70.9% (63.0-78.0)  **Positive predictive value:** 58.6% (44.7-67.8)  **Negative predictive value**: 95.2% (89.3-98.0)  *The study did not present data that would allow the calculation of the positive and negative likelihood ratio with their respective confidence intervals. |
| CONCEIÇÃO et al.,  2021  Bahia – Brazil  ***Journal of Periodontology*** | To evaluate the  diagnostic criteria used in a variety of epidemiological studies of periodontitis in pregnant women.  **Funding sources**: Government institutions. | Cross-sectional study  **Sample**: 671 pregnant women | Diagnosis according to  (CDC/AAP):  1. Severe periodontitis: ≥ 2 interproximal sites with  cliCALal attachment level (CAL) ≥ 6 mm (in different teeth) and ≥ 1 interproximal site with probing depth (PS) ≥ 5 mm.  2. Moderate periodontitis:  ≥ 2 interproximal sites with CAL ≥ 4 mm (in different teeth) or ≥ 2 interproximal sites with PS ≥ 5 mm. 3. Mild periodontitis: ≥2 interproximal sites with CAL ≥ 3 mm (not on the same tooth) and ≥2 interproximal sites with PS ≥4 mm (not on the same tooth) or 1 site with PS ≥ 5 mm.  4. No periodontitis: no “mild” or “moderate” or “severe” periodontitis | Diagnosis according to  **Gomes-Filho et al. 2018**:  1. Severe periodontitis  ≥ 2 teeth with ≥ 1 sites with PS ≥ 5 mm, with  CAL ≥ 5 mm and bleeding on probing in the same CAL.  2. Moderate periodontitis: ≥ 2 teeth with ≥ 1 sites with PS ≥ 4 mm, with CAL ≥ 3 mm  and bleeding on probing at the same site 3. Mild periodontitis: ≥ 2 teeth with ≥ 1 sites with PS ≥ 4 mm, with CAL ≥ 1 mm and bleeding on probing at the same site.  4. No periodontitis: absence of “mild” or “moderate” or “severe” periodontitis.  Diagnosis according to  **Albandar et al. 2007:**  1. Severe periodontitis  ≥ 2 teeth with ≥ 1 sites with PS ≥ 5 mm, with  CAL ≥ 5 mm.  2. Moderate periodontitis: ≥ 2 teeth with ≥ 1 PS sites  ≥ 4 mm and CAL ≥ 5 mm. 3. Mild periodontitis: ≥ 1 tooth with ≥ 1 sites with PS ≥ 4 mm and CAL ≥ 4 mm.  4. No periodontitis: absence of “mild” or “moderate” or “severe” periodontitis.  Diagnosis according to  **Bassani et al. 2007:**  1. Severe periodontitis  ≥ 3 interproximal  locations (on different teeth)  with CAL ≥ 7 mm. 2. Moderate periodontitis: ≥ 3 interproximal sites (in  different teeth) with CAL 5 mm. 3. Mild periodontitis: ≥ 3  interproximal sites (in  different teeth)  with CAL ≥ 3  mm. 4. No periodontitis: absence of “mild” or “moderate” or “severe” periodontitis.  Diagnosis according to  **Lopez et al. 2002:**  - Periodontitis: ≥ 4 teeth with ≥ 1 sites with PS  ≥ 4 mm, and with CAL ≥ 3  mm on a website.  Diagnosis according to  **Nesse et al. 2008:**  - Periodontitis: Individuals  with the highest PISA quartile  amount. | **Occurrence of periodontitis:**  CDC/AAP: 63.6% (n=445) | **Occurrence of periodontitis:**  Gomes-Filho et al. 2018: 31.0% (n=208)  Albandar et al. 2007:  43.1% (n=289)  Bassani et al. 2007: 91.9% (n=610)  Lopez et al. 2002: 25.0%  (n=168)  Nesse et al. 25.0% (n=168) | Gomes-Filho et al. 2018  **Sensitivity**: 46.7% (42.0-51.5)  **Specificity**: 100% (98.4-100)  **Positive predictive value**: 55.8% (48.7- 62.6)  **Negative predictive value**: 100% (99.2-100)  **Positive likelihood ratio**: 6.03 (5.01- 7.27)  **Negative likelihood ratio**: 0  Albandar et al. 2007  **Sensitivity**: 62.9% (58.2-67.4)  **Specificity**: 96.0% (92.6-98.2)  **Positive predictive value**: 96.9% (94.2-98.6)  **Negative predictive value**: 56.8% (51.7-61.8)  **Positive likelihood ratio**: 15.8 (8.3- 30.1)  **Negative likelihood ratio**: 0.38 (0.34-0.43)  Bassani et al. 2007  **Sensitivity**:98.2% (96.5-99.2)  **Specificity**:23.5% (18.1-29.5)  **Positive predictive value**:71.6% (67.9-75.2)  **Negative predictive value**:81.9% (75.8-94.2)  **Positive likelihood ratio**: 1.28 (1.19-1.38)  **Negative likelihood ratio**: 0.07 (0.03-0.15)  Lopez et al. 2002  **Sensitivity**: 37.3% (32.8-42.0)  **Specificity**: 99.1% (96.8-99.9)  **Positive predictive value:98.8% (95.8-99.9)**  **Negative predictive value**:44.5% (40.1-49.0)  **Positive likelihood ratio**: 42.2 (10.6-168)  **Negative likelihood ratio**:0.63 (0.58-0.68)  Nesse et al. 2008  **Sensitivity**: 36.0% (31.5-40.6)  **Specificity**: 96.5% (93.1-98.5)  **Positive predictive value**:95.2% (90.8-97.9)  **Negative predictive value**: 43.3% (39.0-47.8)  **Positive likelihood ratio**: 10.2 (5.09-20.3)  **Negative likelihood ratio**: 0.66 (0.61-0.71) |
| MICU et al.,  2020  Romania  ***Plos One*** | To develop, validate its internal consistency and evaluate a self-reported periodontitis instrument and compare it to cliCALal periodontal diagnosis in a group of postpartum women.  **Funding sources**: Government institutions-  University of MediCALe and Pharmacy "Iuliu Hațieganu". | Cross-sectional study  **Sample**: 215  pregnant women | Diagnosis according to  (CDC/AAP):  1. Severe periodontitis: ≥ 2 interproximal sites with  cliCALal attachment  level (CAL) ≥ 6 mm (in different teeth) and ≥ 1 interproximal site with probing depth (PS) ≥ 5 mm.  2. Moderate periodontitis:  ≥ 2 interproximal sites with CAL ≥ 4 mm (in different teeth) or ≥ 2 interproximal sites with PS ≥ 5 mm.  3. Mild periodontitis: ≥2 interproximal sites with CAL ≥ 3 mm (not on the same tooth) and ≥2 interproximal sites with PS ≥4 mm (not on the same tooth) or 1 site with PS ≥ 5 mm.  4. No periodontitis: no “mild” or “moderate” or “severe” periodontitis. | Self-reported periodontitis: A 16-item questionnaire was used to obtain information about perceived periodontal changes (9 items) and CAL hygiene habits (7 items). The self-reported periodontitis symptoms section of the questionnaire contained 9 questions and was created by 3 native-speaking Romanian periodontists. | **Occurrence of periodontitis:**  **CDC/AAP:** 77.6% (n=167) | Gum swelling: 27.9% (n=60)  Halitosis: 20.9% (n=45)  Previous diagnosis of periodontitis: 13.4% (n=29)  Previous treatment of periodontitis: 8.8% (n=19) | Gum swelling:  **Sensitivity:** 31.1% (24.1-38.2)  **Specificity:** 83.3% (72.8-93.9)  **Positive predictive value:** 86.7% (78.1-95.3)  **Negative predictive value:** 25.8% (18.9-32.7)  **Positive likelihood ratio:** 1.87 (0.95-3.66)  **Negative likelihood ratio:**0.83 (0.70-0.97)  Halitosis:  **Sensitivity:** 24.0% (17.5-30.4)  **Specificity:** 89.6% (80.9-98.2)  **Positive predictive value:** 88.9% (79.7-98.1)  **Negative predictive value:**25.3% (18.8-31.8)  **Positive likelihood ratio:**2.30 (0.96-5.50)  **Negative likelihood ratio:**0.85 (0.75-0.97)  Previous diagnosis of periodontitis:  **Sensitivity:**16.8% (11.1-22.4)  **Specificity:**97.9% (93.9-100)  **Positive predictive value:**96.6% (89.9-100)  **Negative predictive value:**25.3% (19.0-31.5)  **Positive likelihood ratio:** 8.05 (1.12-57.63)  **Negative likelihood ratio:** 0.85 (0.79-0.92)  Previous treatment of periodontitis:  **Sensitivity:** 10.8% (6.1-15.5)  **Specificity:** 99.9% (93.9-100)  **Positive predictive value:**94.7% (87.7-100)  **Negative predictive value:**24.0% (18.0-30.0  **Positive likelihood ratio:** 5.17 (0.71-37.77)  **Negative likelihood ratio:** 0.91 (0.85-0.97) |
| TURTON, HENKEL E ÁFRICA  2017  South Africa  ***Biomarkers*** | Inform obstetricians and other healthcare professionals in antenatal cliCALs about the value of BANA (N-benzoyl-DL-arginine-2-naphthylamide) as a point-of-care test (outside of the dental setting) to detect potential periodontal pathogens and reduce the risk of adverse pregnancy outcomes due to periodontitis.  **Funding sources**: National Research Foundation (NRF) of South Africa. | Cross-sectional study  **Sample**: 443  pregnant women | The presence and severity of periodontal disease were classified as absent, mild, moderate or severe, as described by Offenbacher et al. (2001):  1. Mild periodontitis: ≥ 3 mm or CAL ≥ 2 mm  2. Moderate periodontitis: two or more sites with PS ≥ 5 mm and two or more sites with CAL ≥ 2 mm    3. Severe periodontitis: four or more sites with PS ≥ 5 mm and four or more sites with CAL ≥ 2 mm.  4. Without periodontitis: PD <3 mm and CAL <2 mm | For the BANA assay, a sample of interdental subgingival plaque was collected with a periodontal probe between the first molar and second premolar of each quadrant of the mouth, or, if one of these teeth was missing, between the first and second molars, or between the premolars. The sample collection location was recorded on the BANA test card (Perioscan®, Oral-B Laboratories Inc., Redwood City, CA) in the marked space. Briefly, the test principle is as follows. The BANA Hydrolysis Test is a plastic card with two separate reagent matrices (strips). The lower strip is impregnated with BANA reagent and the upper strip contains a chromogenic diazo reagent, Fast Black K B-naphthylamide. One of the hydrolytic products of the BANA reaction reacts with Fast Black K producing a permanent blue color.  The result of the BANA analysis was recorded as either positive (blue spots on the sample CAL on the reagent card) or negative (no color change) on the data capture sheet. | **Occurrence of periodontitis**:  Offenbacher et al. (2001): 9.93% (n=44) | **Occurrence of periodontitis**:  BAN: 64%  (n=282) | **Sensitivity:** 86.25% (82.04-89.06)  **Specificity:** 95.12% (89.77-97.75)  **Positive predictive value:** 97.87% (95.44-99.02)  **Negative predictive value:** 72.67% (65.32-78.97)  **Positive likelihood ratio:** 17.68 (12.74-24.54)  **Negative likelihood ratio:** 0.14 (0.13-0.15) |
| KUGAHARA, SHOSENJI E OHASHI,  2008  Japan  ***Journal of Obstetrics and Gynaecology*** | Develop a test for screening pregnant women for periodontitis using saliva before a dental examination  **Funding so**urces: not reported. | Cross-sectional study  **Sample**: 221  pregnant women | Diagnosis according to  Community Periodontal Treatment Needs Index (CPITN):  healthy periodontium (CPITN 0), gingivitis (CPITN score 1, 2), and periodontitis (CPITN 3, 4). | Salivary enzymatic test: lactate dehydrogenase (LDH), aspartate aminotransferase (AST) and alkaline phosphatase (ALP) + occult blood in saliva. The screening test indicated a positive result when LDH = 684 IU/L, ALP = 75 IU/L and/or positive occult blood were observed.  Occult blood test: To determine the amount of occult blood in saliva, Salivaster-Bld (Showa Yakuhin Kako, Tokyo, Japan) was used. The test procedure involved dipping the test paper in saliva and then comparing it to a standard color chart. | **Occurrence of periodontitis:**  CPITN: 8.6% (n=19) | **Occurrence of periodontitis:**  Salivary enzyme test: 7.7% (n=17) | **Sensitivity:** 89.47% (68.91-67.06)  **Specificity:** 62.38% (55.52-68.67)  **Positive predictive value:**18.28% (11.74-27.34)  **Negative predictive value:** 98.44% (19.0-31.5)  **Positive likelihood ratio**: 2.37 (2.28-2.47)  **Negative likelihood ratio:** 0.16 (0.06-0.45) |
