# Supplementary material for: Systematic review to evaluate accuracy studies of the diagnostic criteria for periodontitis in pregnant women
Source: PLoS One. 2024 Jul 17;19(7):e0304758. doi: 10.1371/journal.pone.0304758 (PMC11253960; doi:10.1371/journal.pone.0304758)
Supplement: S3 File — (DOCX) [file pone.0304758.s004.docx]

**S3 Chart 3.** List of excluded studies and reason for exclusion.

| **STUDY** | | **REASON FOR EXCLUSION** |
| --- | --- | --- |
| 1 | Chiga S, Ohba T, Tanoue D, Kawase H, Katoh T, Katabuchi H. [Validity of Self-Reported Periodontal Disease Questionnaire among Pregnant Women]. Nihon Eiseigaku Zasshi. 2016;71(3):260-266. Japanese. doi: 10.1265/jjh.71.260. PMID: 27725429. | Incomplete outcome. |
|  |  |  |
| 2 | Al Habashneh R, Khader YS, Jabali OA, Alchalabi H. Prediction of preterm and low birth weight delivery by maternal periodontal parameters: receiver operating characteristic (ROC) curve analysis. Matern Child Health J. 2013 Feb;17(2):299-306. doi: 10.1007/s10995-012-0974-2. PMID: 22392602. | No outcomes of interest. |
|  |  |  |
