# Supplementary material for: Systematic review to evaluate accuracy studies of the diagnostic criteria for periodontitis in pregnant women
Source: PLoS One. 2024 Jul 17;19(7):e0304758. doi: 10.1371/journal.pone.0304758 (PMC11253960; doi:10.1371/journal.pone.0304758)
Supplement: Attachment — Submitted filename: Responses to Reviewers - 10 05 2024.docx [file pone.0304758.s006.docx]

***Dear Erika Barbara Abreu Fonseca Thomaz, Ph.D***

**Academic Editor**

**PLOS ONE**

Provided below are the responses to the reviewers’ comments to the manuscript, **“Systematic Review to Evaluate Accuracy Studies of the Diagnostic Criteria for Periodontitis in Pregnant Women”** (PONE-D-23-35573R1).

Journal Requirements:

Please review your reference list to ensure that it is complete and correct. If you have cited papers that have been retracted, please include the rationale for doing so in the manuscript text, or remove these references and replace them with relevant current references. Any changes to the reference list should be mentioned in the rebuttal letter that accompanies your revised manuscript. If you need to cite a retracted article, indicate the article’s retracted status in the References list and also include a citation and full reference for the retraction notice.

**Response:** The reference list was reviewed, as suggested.

**ADDITIONAL EDITOR COMMENTS**

Dear authors,

After carefully reading this new version, I consider that the decisions made by the authors made the manuscript more appropriate. I only have a small observation regarding the two new paragraphs inserted at the end of the Introduction, as mentioned below.

Please, I recommend that you remove the names of the authors of the sentence. Leave only the numerical references. This will make the text cleaner and easier for the reader to understand. The various "commas", "semicolons" and "and" throughout the sentence make the text very confusing. I also suggest that the last paragraph of the Introduction (consisting of just 1 sentence of 2 lines) be joined to the previous paragraph, as follows:

"Different criteria used for diagnosing periodontitis in pregnant women have been reported (8-15). Which of these is the best criterion to diagnose periodontitis in pregnant women has not been agreed upon; however, they are important to estimate the frequency of maternal periodontitis and, thus, evaluate the effectiveness of preventive or therapeutic procedures and to determine individual risk (16,17). This systematic review evaluated the accuracy of the criteria for diagnosing periodontitis in pregnant women."

**Response:** As recommended, the two last paragraphs at the end of the Introduction section were rewritten, as follows:

**“INTRODUCTION**

**…**

Different criteria used for diagnosing periodontitis in pregnant women have been reported (8-15). Which of these is the best criterion to diagnose periodontitis in pregnant women has not been agreed upon; however, they are important to estimate the frequency of maternal periodontitis and, thus, evaluate the effectiveness of preventive or therapeutic procedures and to determine individual risk (16,17). This systematic review evaluated the accuracy of the criteria for diagnosing periodontitis in pregnant women.**”**

PONE-D-23-35573R1

Systematic Review to Evaluate Accuracy Studies of the Diagnostic Criteria for Periodontitis in Pregnant Women

PLOS ONE

Dear Dr. Gomes-Filho,

Thank you for submitting your manuscript to PLOS ONE. After careful consideration, we feel that it has merit but does not fully meet PLOS ONE’s publication criteria as it currently stands. Therefore, we invite you to submit a revised version of the manuscript that addresses the points raised during the review process.

After carefully reading this new version, I consider that the decisions made by the authors made the manuscript more appropriate. I only have a small observation regarding the two new paragraphs inserted at the end of the Introduction, as mentioned below.

Please submit your revised manuscript by Jun 23 2024 11:59PM. If you will need more time than this to complete your revisions, please reply to this message or contact the journal office at plosone@plos.org. When you're ready to submit your revision, log on to https://www.editorialmanager.com/pone/ and select the 'Submissions Needing Revision' folder to locate your manuscript file.

Please include the following items when submitting your revised manuscript:

A rebuttal letter that responds to each point raised by the academic editor and reviewer(s). You should upload this letter as a separate file labeled 'Response to Reviewers'.

A marked-up copy of your manuscript that highlights changes made to the original version. You should upload this as a separate file labeled 'Revised Manuscript with Track Changes'.

An unmarked version of your revised paper without tracked changes. You should upload this as a separate file labeled 'Manuscript'.

We look forward to receiving your revised manuscript.

Kind regards,

Erika Barbara Abreu Fonseca Thomaz, Ph.D

Academic Editor

PLOS ONE

Journal Requirements:

Please review your reference list to ensure that it is complete and correct. If you have cited papers that have been retracted, please include the rationale for doing so in the manuscript text, or remove these references and replace them with relevant current references. Any changes to the reference list should be mentioned in the rebuttal letter that accompanies your revised manuscript. If you need to cite a retracted article, indicate the article’s retracted status in the References list and also include a citation and full reference for the retraction notice.

Additional Editor Comments:

Dear authors,

Please, I recommend that you remove the names of the authors of the sentence. Leave only the numerical references. This will make the text cleaner and easier for the reader to understand. The various "commas", "semicolons" and "and" throughout the sentence make the text very confusing. I also suggest that the last paragraph of the Introduction (consisting of just 1 sentence of 2 lines) be joined to the previous paragraph, as follows:

"Different criteria used for diagnosing periodontitis in pregnant women have been reported (8-15). Which of these is the best criterion to diagnose periodontitis in pregnant women has not been agreed upon; however, they are important to estimate the frequency of maternal periodontitis and, thus, evaluate the effectiveness of preventive or therapeutic procedures and to determine individual risk (16,17). This systematic review evaluated the accuracy of the criteria for diagnosing periodontitis in pregnant women."

[Note: HTML markup is below. Please do not edit.]

[NOTE: If reviewer comments were submitted as an attachment file, they will be attached to this email and accessible via the submission site. Please log into your account, locate the manuscript record, and check for the action link "View Attachments". If this link does not appear, there are no attachment files.]

While revising your submission, please upload your figure files to the Preflight Analysis and Conversion Engine (PACE) digital diagnostic tool, https://pacev2.apexcovantage.com/. PACE helps ensure that figures meet PLOS requirements. To use PACE, you must first register as a user. Registration is free. Then, login and navigate to the UPLOAD tab, where you will find detailed instructions on how to use the tool. If you encounter any issues or have any questions when using PACE, please email PLOS at figures@plos.org. Please note that Supporting Information files do not need this step.
